# Unexpected effect of a *Bacteroides* conjugative transposon, CTnDOT, on chromosomal gene expression in its bacterial host

**DOI:** 10.1111/j.1365-2958.2007.05756.x

**Published:** 2007-06-01

**Authors:** Kyung Moon, Justin Sonnenburg, Abigail A Salyers

**Affiliations:** 1 Laboratory of Molecular Biology, National Cancer Institute, National Institutes of Health Bethesda, MD 20892, USA.; 2 Center for Genome Sciences, Washington University School of Medicine St. Louis, MO 63108, USA.; 3 Department of Microbiology, University of Illinois Urbana, IL 61801, USA.

## Abstract

Foreign DNA elements such as plasmids and conjugative transposons are constantly entering new bacterial hosts. A possible outcome of such events that has not been considered previously is that regulatory genes carried on some of them might affect the expression of chromosomal genes of the new host. To assess this possibility, we investigated the effect of the *Bacteroides* conjugative transposon CTnDOT on expression of chromosomal genes in *Bacteroides thetaiotaomicron* 5482 (BT4001). Most of the upregulated genes were genes of unknown function, but a number of them were associated with a region of the chromosome that contained a putative conjugative transposon, which had been tentatively designated as CTn4-bt. Upregulation of CTn4-bt genes and other chromosomal genes affected by CTnDOT was controlled by two regulatory genes on CTnDOT, *rteA* and *rteB*, which encode a two-component regulatory system. Transfer of CTn4-bt was also mediated by *rteA* and *rteB.* Three other putative CTns, CTn1-bt, CTn2-bt and CTn3-bt, were mobilized by CTnERL, a CTn closely related to CTnDOT, but genes from CTnERL other than *rteA* and *rteB* were also required. Unexpectedly, homologous recombination was required for CTn1-bt, CTn2-bt, CTn3-bt and CTn4-bt to integrate in the recipient. Our results show that regulatory genes on an incoming mobile element can have multiple effects on its new host, including the activation of previously non-transmissible elements.

## Introduction

Transfer of DNA across bacterial species and genus lines appears to occur readily in the human and animal intestinal tract (Salyers, 1993; Nikolich *et al*., 1994; Shoemaker *et al*., 2001; Salyers *et al*., 2004). Such broad host range transfers are usually mediated by conjugation and involve a variety of self-transmissible and mobilizable elements such as plasmids and conjugative transposons. Conjugative transposons (CTns), which are integrated elements that excise and transfer by conjugation, seem to be particularly important mediators of gene transfer among species of the numerically predominant genus *Bacteroides* (Shoemaker *et al*., 2001). In the past, studies of CTn-mediated transfer events have focused on the transfer of genes such as antibiotic resistance genes that confer new traits on the recipient strain. Yet, some of these CTns also carry regulatory genes that control such CTn functions as excision and transfer (Stevens *et al*., 1993). What has not been considered previously is the possibility that these regulatory genes might have global effects on the genes of the bacterial strain that acquires them.

It is well established that CTns and self-transmissible plasmids can interact with unlinked elements such as other plasmids or integrated elements by providing transfer proteins *in trans* (Stevens *et al*., 1990; Shoemaker *et al*., 1993). A different question is whether regulatory genes on mobile elements can act on genes outside the mobile element by altering their expression and, if so, what types of impact this might have on the recipient strain. Microarray analysis has now made it possible for the first time to ask whether such global regulatory effects can occur.

We decided to test the effect of a broad host range *Bacteroides* CTn, CTnDOT, on expression of chromosomal genes in the *Bacteroides* strain *Bacteroides thetaiotaomicron* 5482 (designated BT4001; Xu *et al*., 2003). BT4001 is one of the few *Bacteroides* strains for which a genome sequence is available and is the first *Bacteroides* strain for which a microarry has been constructed. The genome of BT4001 does not contain any proven CTns, but annotators had identified four possible cryptic CTns, based mainly on homology of genes in their vicinity to transfer genes of CTnDOT. We were interested in the possibility that these cryptic CTns might be transmissible and might interact with CTnDOT.

*Bacteroides* spp. such as *B. thetaiotaomicron* are of interest not only because they comprise a substantial portion of the normal colonic microflora, about 20–30%, but also because they are significant opportunistic pathogens, which can cause septicaemia and abscesses in almost any organ of the body (Gorbach and Bartlett, 1974a, b, c; Moore *et al*., 1978; Bartlett, 1983). CTnDOT was chosen because it has now become widespread among *Bacteroides* spp. in the human colon, indicating that it is actively transferring in the colonic environment. Also, it carries regulatory genes (Stevens *et al*., 1993).

In addition to two antibiotic resistance genes, *tetQ* and *ermF*, CTnDOT carries three regulatory genes, *rteA*, *rteB* and *rteC.* RteA and RteB form a two-component regulatory system. RteB activates expression of *rteC* (Moon *et al*., 2005), and RteC, in turn, controls the expression of CTnDOT excision and transfer genes. RteA, RteB and RteC are only produced when BT4001-containing CTnDOT is exposed to the antibiotic tetracycline. Accordingly, we were interested in the possibility that one or more of these regulatory genes might exert an influence on expression of genes outside of CTnDOT.

## Results

### Identification of chromosomal genes upregulated in the presence of CTnDOT

The first step in our analysis was to use a recently created GeneChip microarray that carries BT4001 genes to assess the overall effects on chromosomal gene expression of the presence of CTnDOT in the strain. These GeneChips, whose availability is quite limited at present, were the generous gift of Jeff Gordon (Washington University, St Louis, MO). As only two GeneChips were available to us, we used them to assess the pattern of gene expression in two strains and conditions that represented the two extremes of possible effects of CTnDOT on BT4001 gene expression. The strains were BT4001 and BT4001-carrying CTnDOT (BT4007). BT4007 was exposed to tetracycline (1 μg per ml), whereas BT4001 was not. The plan was to use this information to identify genes of interest, whose expression could then be monitored more precisely by quantitative reverse transcription polymerase chain reaction (RT-PCR).

We chose a sevenfold increase in gene expression as our cut-off for changes in gene expression that might be of interest. We had two reasons for this choice. First, as already mentioned, we were only able to obtain one GeneChip to monitor each condition. That is, we did not have access to sufficient numbers of GeneChips to detect reliably small changes in gene expression. Second, tetracycline has been reported to stabilize some messages, an effect that is generally less than five- to sevenfold (Wei and Bechhofer, 2002), so focusing on genes whose expression was higher than this made it more likely that we would find genes affected by CTnDOT regulatory genes as opposed to genes whose expression was influenced by tetracycline on the cell. The strategy was to identify possible genes of interest, which could then be studied further by quantitative RT-PCR.

The results of this preliminary analysis indicated that expression of many genes was affected by the presence of CTnDOT. A list of the genes that exhibited the highest fold increase in expression is provided in Table 1. Most of these genes had been annotated as genes of unknown function. However, the annotators also made some tentative identifications of possible cryptic CTns, based mainly on genes with sequence similarity to transfer genes from CTnDOT (see Table 1). The ends of these putative CTns and other genes that might be part of them could not be identified by sequence similarity with known CTns. Also, there was no evidence that these putative CTns were mobile. Nonetheless, the annotators not only called them CTns but even gave them names such as ‘CTn4’ (Xu *et al*., 2003). As some of our genes of unknown function were linked to the putative CTn4, we decided to focus on these genes.

**Table 1:** List of possible induced chromosomal genes identified by GeneChip analysis.

Proposed function	Possible identity^a^	Fold change^b^	ORF ID^c^
Hypothetical transfer genes of CTn4-bt	*tra I* (fragment)	148	BT4772
	*tra G* (fragment)	74	BT4769
	*tra E*	43	BT4767
	*tra I* (fragment)	35	BT4773
	*tra F*	25	BT4768
	*tra G* (fragment)	25	BT4770
	*tra K* (fragment)	20	BT4776
	*tra J*	18	BT4774
	*tra N*	16	BT4780
	*tra K* (fragment)	12	BT4775
	*tra M* (fragment)	9	BT4779
	*tra A* (fragment)	9	BT4761
	*tra A* (fragment)	8	BT4762
	*tra M* (fragment)	7	BT4778
Integrase/transposase	Tyrosine recombinase	15	BT3135
	Transposase	8	BT1138
Membrane proteins	OmpA homologue	13	BT0066
	Transmembrane protein	9	BT2119
	Polysaccharide export protein	8	BT0060
	α-Galactosidase precursor	10	BT0065
	Aminotransferase	11	BT3737
Chaperone	GroE	7	BT1243
Hypothetical proteins tentatively classified by chromosomal location	CTn4-bt	102	BT4765
	CTn4-bt	78	BT4764
	CTn4-bt	148	BT4766
	CTn4-bt	20	BT4777
	CTn4-bt	8	BT4771
	Other	36	BT0020
	Other	17	BT0064
	CTn4-bt	16	BT4780
	Other	13	BT1073
	Other	14	BT0018
	Other	11	BT0019
	Other	8	BT0059
	Other	7	BT0057
	Other	7	BT0068

a Proposed function and possible identity were determined from database search results. CTn4-bt is a putative CTn identified from the genome sequence analysis. The transfer (*tra*) genes listed are similar to *tra* genes found on the known CTn, CTnDOT. In the case of the hypothetical proteins, the ones encoded by genes located on CTn4 are indicated. ‘Other’ means hypothetical genes not located on CTn4-bt.

b This list was compiled from results of the GeneChip assay using RNA samples from wild-type *B. thetaiotaomicron* 5482 (BT4001) and the same strain containing CTnDOT (BT4007). Fold change was obtained by dChip analysis.

c Location of the open reading frame in the BT4001 genome sequence.

To add to the nomenclatural confusion, annotators of other *Bacteroides* genome sequences, such as the *Bacteroides fragilis* sequences, have followed this example and have identified putative CTns, which have also been given numbers like ‘CTn4’, even though they have no similarity to the ‘CTn4’ of BT4001 (Kuwahara *et al*., 2004). To make clear which species the numbered putative CTn comes from, we will label the putative CTns in the *B. thetaiotaomicron* (BT4001) genome sequence with a bt, e.g. CTn4-bt.

In the genome sequence analysis of BT4001, three other possible CTns were also identified in the same way as CTn4-bt (CTn1-bt, CTn2-bt and CTn3-bt), but none of the genes identified by our microarray analysis were associated with these three putative CTns. Per cent amino acid identities between CTnDOT gene products and predicted gene products from possible homologues encoded by CTn1-CTn4-bt are provided in Table 2. Many of them were as similar in certain regions as CTn4-bt to CTnDOT, but were apparently not similar enough to be activated by CTnDOT.

**Table 2:** Comparison of percentage amino acid identity between Tra proteins from CTnDOT and Tra proteins encoded on the four putative conjugative transposons in the *B. thetaiotaomicron* 5482 chromosome.

Transfer protein	Putative cryptic CTns in the chromosome			
	
CTnDOT	CTn1-bt	CTn2-bt	CTn3-bt	CTn4-bt
TraA	57	63	40	43
TraB	27	35	20	38
TraC	29	27	NA^a^	28
TraD	NA	24	NA	NA
TraE	93	88	85	89
TraF	71	66	64	63
TraG	73	69	68	69
TraH	36	21	70	NA
TraI	65	65	67	61
TraJ	75	59	72	64
TraK	71	71	77	68
TraM	48	43	45	34
TraN	55	64	65	54
TraO	59	54	52	36
TraQ	43	38	50	NA

a NA is not available because of the lack of the target gene within the CTn. The transfer protein on CTnDOT is shown in the first column and is compared with homologues found encoded on the four cryptic CTns in the *B. thetaiotaomicron* 5482 chromosome.

Quantitative RT-PCR was used to measure more precisely the changes in expression of these selected genes when CTnDOT was present and cells were stimulated by tetracycline. CTnDOT integration was stable throughout the duration of the experiment. Results of this analysis are summarized in Table 3. Expression of several genes not associated with CTn4-bt was also tested to assess further whether the results of the preliminary GeneChip comparison were likely to be reliable. Expression changes of most of these genes agreed with the preliminary GeneChip results (Table 3).

**Table 3:** Fold induction of selected chromosomal genes of BT4001 affected by CTnDOT, as measured by quantitative RT-PCR.^a^

ORF ID	Proposed function	BT4001ΩQAB^b^	BT4007	BT4001(pRteC)^b^
BT4762	TraA (CTn4-bt)	30 (± 13)	143 (± 21)	5 (± 2)
BT4763	TraC (CTn4-bt)	14 (± 2)	44 (± 12)	3 (± 2)
BT4764	HP (CTn4-bt)	96 (± 38)	95 (± 16)	2 (± 1)
BT4766	HP (CTn4-bt)	45 (± 13)	163 (± 18)	1 (± 1)
BT4767	TraE (CTn4-bt)	121 (± 23)	257 (± 25)	1 (± 1)
BT4768	TraF (CTn4-bt)	216 (± 10)	350 (± 17)	3 (± 1)
BT4770	TraG (CTn4-bt)	330 (± 13)	531 (± 26)	1 (± 1)
BT4780	TraN (CTn4-bt)	1 (± 9)	116 (± 17)	1 (± 0)
BT0018	HP^c^	22 (± 3)	30 (± 10)	2 (± 1)
BT0019	HP	24 (± 4)	29 (± 4)	2 (± 1)
BT0020	HP	60 (± 9)	66 (± 21)	2 (± 1)
BT0022	HP	9 (± 1)	33 (± 11)	1 (± 1)
BT0060	Polysaccharide export protein	1 (± 1)	32 (± 7)	1 (± 1)
BT0065	α-Galactosidase precursor	42 (± 12)	29 (± 2)	1 (± 1)
BT3135	Integrase	19 (± 8)	16 (± 4)	1 (± 1)
BT2118	Transporter (AcrBDF family)	39 (± 0)	7 (± 0)	1 (± 0)

a The fold induction was obtained by quantitative RT-PCR analysis. The internal standard, a constitutively expressed single-copy gene, was the σ^70^ gene. Triplicate experiments were analysed in three independent experiments and the results were described as fold induction (±SD). Fold induction (*N*) was calculated by the following formula: *N* = 2^ΔΔCt^ = 2(^ΔCt target-ΔCt σ^70^^), where ΔΔCt is ΔCt target − ΔCt σ^70^ and ΔCt is the difference in threshold cycles for the target and the σ^70^ reference.

b BT4001ΩQAB is BT4001 containing a single copy of the *tetQ-rteA-rteB* operon. BT4007 is BT4001 containing a copy of CTnDOT. BT4001(pRteC) is BT4001 containing a copy of pRteC, a plasmid containing *rteC* in which *rteC* is expressed constitutively from a heterologous promoter (Moon *et al*., 2005).

c HP, hypothetical proteins which do not have any homologues in the databases.

### Genes on CTnDOT responsible for the regulatory effect

We examined the genes listed in Table 3 in more detail to determine which CTnDOT genes were responsible for the increased expression of the BT4001 chromosomal genes. To do this, we compared expression of the genes shown in Table 3 in strains of BT4001 that contained only *tetQ, rteA* and *rteB* (BT4001ΩQAB) with that in strains that contained the entire CTnDOT (BT4007). In both cases, cells were stimulated by tetracycline to trigger the production of RteA and RteB.

In all but two cases, the CTnDOT central regulatory genes, *rteA* and *rteB*, alone accounted for the increase in gene expression. In the other two cases, increases in expression were higher in cells containing the entire CTnDOT than they were in the strain that only contained *rteA* and *rteB*, although *rteA* and *rteB* still accounted for a significant part of the upregulation.

As we used tetracycline stimulation to trigger production of RteA and RteB, the question arose as to whether tetracycline alone might be responsible for the effect. To test this, and to test a possible effect of RteA alone, we tested expression of the genes listed in Table 3 in a strain of BT4001 that contained only *tetQ* and *rteA* (BT4001ΩQA). There was no stimulation of expression of any of the genes in this strain (data not shown). Thus, neither tetracycline nor *rteA* alone had any effect. The *rteB* gene was needed.

Previous studies of CTnDOT excision and transfer had shown that a third regulatory gene, *rteC*, whose expression is controlled by RteA/RteB, is required for expression of CTn excision and transfer genes (Stevens *et al*., 1993; Li *et al*., 1995; Cheng *et al*., 2001). In fact, a form of *rteC* had been constructed in which the normal promoter was replaced by a constitutively expressed promoter. This form of *rteC* could control expression of the CTnDOT excision and transfer genes in the absence of *rteA* and *rteB* (Moon *et al*., 2005). Accordingly, we tested whether RteC played a role in the expression of chromosomal genes by monitoring expression of these genes in strain BT4001pRteC, which contained a copy of the plasmid that constitutively expressed RteC. Control by RteC was not seen in the case of any of the upregulated BT4001 chromosomal genes we tested (Table 3). Thus, *rteA* and *rteB* seem to be the regulatory genes mainly responsible for the changes in gene expression we observed.

### Transfer of CTn4-bt and other putative CTn-bts

The fact that expression of some genes on CTn4-bt was stimulated by tetracycline in the presence of *rteA* and *rteB* raised the possibility that CTnDOT might be rendering CTn4-bt capable of transfer through a regulatory interaction rather than through *in trans* action of transfer proteins. To determine if CTn4-bt was mobilizable by RteA and RteB action, we introduced a selectable marker, *ermG*, into a non-coding region of CTn4-bt. As CTnDOT contains *ermF*, and as the number of selectable markers available for use in *Bacteroides* spp. is limited, we used a closely related CTn, CTnERL, as a stand-in for CTnDOT. CTnERL and CTnDOT are virtually identical at the DNA sequence level, except that CTnDOT has a 13 kb insertion that contains *ermF* (Whittle *et al*., 2001). CTnERL only carries *tetQ.*

The results of this experiment revealed that CTn4-bt was not self-transmissible in the absence of CTnERL but was transferred when CTnERL was present and the cells were exposed to tetracycline (Table 4). Moreover, *rteA* and *rteB* alone were sufficient to stimulate CTn4-bt transfer. That is, CTn4-bt was providing the genes needed for its own transfer but these genes are not expressed unless foreign regulatory genes were provided by CTnERL. This is different from situations, previously reported, in which CTnERL or CTnDOT provides transfer proteins *in trans* that mobilize another element such as a plasmid (Shoemaker *et al*., 1986).

**Table 4:** Transfer of four cryptic CTns and cryptic plasmid p5482A due to CTnERL and/or its regulatory genes, *rteA*, *rteB* and *rteC*.

Strains (BT4001 strains)	–Tc^b^	+Tc^b^
ΩCTnERL^a^ΩCTn4-bt::*ermG*	< 4 × 10^−9^	4 × 10^−6^ to 5 × 10^−6^
ΩCTnERLΩCTn3-bt::*ermG*	< 1 × 10^−8^	2 × 10^−6^ to 5 × 10^−7^
ΩCTnERLΩCTn2-bt::*ermG*	< 1 × 10^−8^	1 × 10^−5^ to 1 × 10^−6^
ΩCTnERLΩCTn1-bt::*ermG*	< 4 × 10^−9^	3 × 10^−6^ to 2 × 10^−7^
ΩQABCΩCTn4-bt::*ermG*	< 4 × 10^−9^	2 × 10^−6^ to 2 × 10^−7^
ΩQABCΩCTn3-bt::*ermG*	< 1 × 10^−8^	< 1 × 10^−8^
ΩQABCΩCTn2-bt::*ermG*	< 1 × 10^−8^	< 1 × 10^−8^
ΩQABCΩCTn1-bt::*ermG*	< 4 × 10^−9^	< 4 × 10^−9^
ΩQABΩCTn4-bt::*ermG*	< 1 × 10^−9^	< 1 × 10^−7^
ΩCTnERL(p5482A)::*ermG*	< 2 × 10^−9^	< 2 × 10^−9^
ΩQABC(p5482A)::*ermG*	< 2 × 10^−9^	< 2 × 10^−9^

a CTnERL is nearly identical to CTnDOT except that CTnDOT has a 13 kb region containing *ermF* between the *oriT* and *int* genes. CTn1-bt to CTn4-bt are indicated explicitly in each strain to show which CTn has the *ermG* insertion, although each strain also contains the other three CTns.

b The numbers represent the transfer frequency, expressed as numbers of transconjugants per recipient cells (BT4001) at the end of the mating with the tetracycline-induced BT4100 donors containing the CTnDOT genes shown in the first column.

Although CTn4-bt could transfer if *rteA* and *rteB* were present, a defect in CTn4-bt became evident when we tested the transfer of CTn4-bt to *B. fragilis* 2553R and *B. fragilis* 638R, rather than to BT4001. CTnERL and CTnDOT both transfer to these *B. fragilis* strains at frequencies comparable to the frequency of their transfer to BT4001 (Table 4). Yet no CTn4-bt transconjugants were obtained when the *B. fragilis* strains were used as recipients. A possible explanation for this result was that CTn4-bt, although put into motion when CTnDOT activated expression of its transfer genes, was defective in its ability to integrate independently of homologous recombination in the recipient.

When BT4001 was the recipient, CTn4-bt could have integrated by homologous recombination because CTn4-bt is part of the recipient BT4001 chromosome, but this type of integration was not possible in the *B. fragilis* strains. To test this possibility, we used as recipient a mutant of BT4001 that was defective in homologous recombination (BT4001 *recA*^–^). This strain contained a single cross-over disruption in the BT4001 *recA* gene. This recipient did not serve as a recipient for CTn4-bt although it contained CTn4-bt genes. Thus, CTn4-bt is presumably defective in its equivalent of this gene or lacks an integrase gene altogether. The apparent lack of a functional integrase was surprising because it raises the question of how CTn4-bt could excise if its integrase was defective. Past studies have shown that CTn integrases are essential for excision (Cheng *et al*., 2000). Thus, if CTn4-bt lacks an active integrase, it is not clear how it excised to initiate the transfer process transfer. As *rteA* and *rteB* alone were sufficient to trigger CTn4 transfer, CTnERL is clearly not needed to provide its integrase *in trans*. It is also possible that CTn4 excised not by an integrase-mediated process but by homologous recombination.

The integrase genes of CTnDOT and CTnERL have proven to be expressed constitutively, but it was possible that the integrase of CTn4-bt was inducible and required RteA and RteB for expression. To test this possibility, we repeated the mating assays with BT4001*recA-* into which the *tetQ-rteA-rteB* operon had been introduced as described for BT4001ΩQAB. As the *tetQ-rteA-rteB* operon was cloned on a deleted form of the mobilizable transposon NBU2, which integrates independent of homologous recombination, it could be introduced into this strain. Both the recipient and the donor were exposed throughout the mating procedure to tetracycline. If RteA and RteB triggered expression of a CTn integrase, which acts independently of homologous recombination, this recipient should allow the integration of CTn4-bt. However, no integration of the transferred element was detected (< 10^−8^ transconjugants per recipient).

Three other possible CTns had been identified during analysis of the BT4001 genome sequence (Xu *et al*., 2003). These were designated CTn1-bt, CTn2-bt and CTn3-bt (Table 2). Results of our microarray analysis did not detect elevated expression of any putative transfer genes associated with CTn1-3-bt. Nor was there any evident change in expression of a possible tyrosine recombinase located near CTn2-bt, which could be the CTn2-bt integrase. Nonetheless, we decided to determine if any of these putative CTns was mobilized by CTnERL. We inserted the *ermG* gene into a non-coding region of each of these CTns and tested them for transfer. Results are shown in Table 4. All three were transferred to BT4001 if CTnERL was present in the donor, but no transfer was detected if CTnERL was absent. As was the case with CTn4-bt, no transfer was detected if the recipient was a *B. fragilis* strain or a homologous recombination-deficient mutant of BT4001. No transfer of CTn1-bt, CTn2-bt or CTn3-bt was detected if only the *tetQ-rteA-rteB* operon was present in the donor. Thus, unlike CTn4-bt, CTn1-3-bt apparently require gene products provided by CTnDOT in addition to RteA and RteB.

### Cryptic plasmid p5482 is not transferred

A cryptic 30 kb plasmid, p5482, is present in BT4001. This plasmid is large enough to be transmissible. As CTn1-bt, CTn2-bt and CTn3-bt were transmissible by CTnERL, even though CTnERL seemed not to control expression of their genes, it was possible that p5482A was also transmissible. To test this possibility, we integrated *ermG* in a non-coding region of this plasmid and tested it for transfer. No transfer was detected (< 10^−8^ transconjugants per recipients) even when the entire CTnDOT was present in the strain. Thus, p5482 appears to be non-transmissible, at least under the conditions we tested.

## Discussion

We have shown for the first time that entry of CTnDOT into a new *Bacteroides* host, BT4001, can have complex consequences, including substantial increases in the expression of a number of chromosomal genes. In this study, we focused on genes whose function we could guess, the genes of a putative CTn, CTn4-bt. Yet, the presence of CTnDOT clearly had more widespread effects on gene expression. In fact, we also detected a number of genes whose expression was downregulated 5- to 65-fold (data not shown). The number of these genes was as large as the number of upregulated genes. The downregulated genes tended not to be genes of unknown function. They included among the most affected genes an ATP-dependent RNA helicase, a type I restriction enzyme, a glycosyltransferase and a homologue of an RNA-binding protein RbpA. Pursuing these genes was, however, beyond the focus of the present study, in particular because there was no discernible pattern in the genes that were affected. It is clear, however, that downregulation as well as upregulation of genes by CTnDOT can occur.

The only other report of this type is the recent finding that the Ti plasmid of *Agrobacterium tumefaciens* was associated with increased production of two proteins of unknown function, which were detected by two-dimensional gel analysis (Lai *et al*., 2006). As an inducer of Ti plasmid genes was necessary for the effect, regulatory genes were probably involved, but the authors of the study did not determine which Ti plasmid genes were required. It is interesting to note that the Ti plasmid, like CTnDOT, has genes for a two-component regulatory system that acts as a central controller of Ti plasmid transfer genes, so this regulatory system might well have been responsible. This possibility was not addressed by the authors. Nonetheless, it may prove to be the fact that many two-component regulatory systems have much wider regulatory effects than are usually thought to be included in a regulon of related genes.

A number of CTn4-bt genes were induced by RteA and RteB. The fact that these genes encoded proteins that are homologues of transfer proteins encoded by CTnDOT genes is consistent with the ability of RteA and RteB to trigger transfer of CTn4-bt. The only other known case in which RteA and RteB are known to induce expression of a gene outside of CTnDOT is an as yet unidentified excision gene on the mobilizable transposon NBU1 (Li *et al*., 1993). In the case of NBU1, however, transfer proteins of CTnDOT were also required for NBU1 mobilization, despite the fact that CTnDOT transfer gene homologues on CTn1-CTn3-bt were as similar to CTnDOT genes as those on CTn4-bt. Yet the genes on CTn1-3-bt were apparently not induced by RteA and RteB. Presumably, the ability of CTnDOT to mobilize these CTns was due solely to the fact that CTnDOT transfer proteins were acting *in trans* to mobilize CTn1-3-bt, a type of mobilization reported widely for CTns and self-transmissible plasmids (Whittle *et al*., 2002a). This finding raises an important cautionary reminder: per cent amino sequence identity does not prove function.

An unexpected feature of CTn1-4-bt was that although DNA from these CTns was transferred to a recipient, integration required homologous recombination. Normally, integrases of CTns catalyse integration in a homology-independent fashion similar to integration catalysed by lambdoid phage integrases. Re-inspection of the genome sequence of BT4001 in the vicinity of CTn1-4-bt revealed no integrase homologue. This does not rule out the presence of an integrase on these CTns because there is great sequence diversity among CTn integrase genes and other integrase genes in the databases. Yet, this observation raises the possibility that these apparent CTns are remnants of CTns that have lost integrase genes. If so, or if the integrases are merely inactive, some integrase must be acting *in trans* because these CTns presumably had to excise in order to transfer and excision requires an integrase.

A possibility we were able to rule out is that the integrase of CTn4-bt was induced by RteA and RteB. However, the presence of the *tetQ-rteA-rteB* operon in the recipient, which should have induced expression of a CTn4-bt integrase, if this hypothesis was correct, was not sufficient to allow integration of CTn4-bt in a recombination-deficient recipient. A third possibility that cannot be ruled out at this point is that all four of these CTns are being transferred by an Hfr type mechanism. Recently, CTnDOT has been shown to be able to transfer like an Hfr if its integrase gene is disrupted (Whittle *et al*., 2006).

Regardless of how CTn1-4-bt DNA is integrated after transfer, parts of it can obviously be preserved in a recipient if homologous recombination with a similar copy of the CTn is present. This could be an explanation for why we have observed considerable variation in CTns that are closely related to CTnDOT. Perhaps the most important implication of our findings, however, is that an incoming mobile element can have a rather substantial effect on expression of genes in a recipient.

The effects of RteA and RteB on BT4001 gene expression required the presence of tetracycline.

This raises the question of how relevant the sort of phenomenon we are reporting here is to a real-world situation. Tetracycline is no longer used as widely as it once was due to the development widespread tetracycline resistance in many species of bacteria. A notable exception is the use of tetracycline to treat skin conditions such as acne or rosacea, treatments that can last for months or years (Jones *et al*., 1989; Sapadin and Fleischmajer, 2006). Tetracycline exposure may actually increase once again in the future. Tetracycline has been considered for use to prevent or treat periodontal disease (Wade *et al*., 1992; Radvar *et al*., 1996; Loesche, 1999). Tetracycline residues have been found in groundwater and water effluent from sewage treatment plants (Hamscher *et al*., 2002; Kummerer, 2004). A new tetracycline derivative, tigecycline, has recently been approved for use and seems to be effective against many bacteria that are resistant to other tetracyclines. Thus, the average person may have a greater and more frequent exposure to tetracycline than might at first appear to be the case.

Given the large number of genes affected by the presence of CTnDOT when the strain that carries it is exposed to tetracycline, it is surprising that CTnDOT type elements are so stably maintained. In fact, about 80% of human colonic *Bacteroides* strains now harbour CTnDOT type elements (Shoemaker *et al*., 2001). This level of carriage has increased from 20% to 30% as the pre-1970 period, so CTnDOT type CTns are not only spreading in the human colon but persisting the in face of tetracycline usage.

In recent years, scientists and physicians have begun to discuss the possible consequences of antibiotic treatment of a patient's disease on the patient's resident bacterial populations. So far, concern has been focused on possible disruptions in the population structure of bacterial microflora and increased selection for resistant strains. Our findings suggest another reason for concern; exposure to an antibiotic could trigger changes in gene expression in some intestinal bacteria that have as yet unknown consequences.

## Experimental procedures

### Bacterial strains, plasmids and growth conditions

Strains and plasmids used in this study are listed in Table 5. BT4007 contains a single copy of CTnDOT in the chromosome. BT4001ΩQABC contains a single copy of the central regulatory region of CTnDOT, *tetQ-rteA-rteB-rteC*, in the chromosome of BT4001 (Whittle *et al*., 2002b). BT4001ΩQAB has a single copy of *tetQ-rteA-rteB* without *rteC*. *Bacteroides* strains were grown in chopped meat medium, and then transferred to TYG (Trypticase-yeast extract-glucose) medium containing tetracycline (1 μg ml^−1^; induced) or no tetracycline (uninduced). BT4100 is a spontaneous thymidine-requiring strain of *B. thetaiotaomicron.* Antibiotic concentrations (in μg ml^−1^) were: ampicillin, 100; cefoxitin, 20; chloramphenicol, 10; erythromycin, 10; gentamicin, 200; and tetracycline, 1, Trimethoprim 100, Thymidine 100.

**Table 5:** Bacterial strains and plasmid used in this study.

Strain or plasmid	Description	Reference or source
Strains		
HB101 (RP1)	HB101 *Escherichia coli* strain containing IncPα plasmid RP1	Shoemaker and Salyers (1987)
*B. fragilis* 2553R	Spontaneous rifampin mutant of *B. fragilis* 2553	Cerdeno-Tarraga *et al*. (2005)
*B. fragilis* 638R	Spontaneous rifampin mutant of *B. fragilis* 638	Smith and Parker (1993)
*B. thetaiotaomicron* 5482A		
BT4001	Spontaneous rifampin mutant of *B. thetaiotaomicron* 5482A	Shoemaker *et al*. (1986)
BT4007	*B. thetaiotaomicron* 4001 that contains wild-type CTnDOT	Shoemaker *et al*. (1989)
BT4100ΩQABC	Spontaneous thymidine-requiring strain of *B. thetaiotaomicron* containing regulatory CTnDOT genes, *tetQ*, *rteA*, *rteB*, *rteC*	Whittle *et al*. (2002b)
BT4001ΩQABC	*B. thetaiotaomicron* containing regulatory CTnDOT genes, *tetQ*, *rteA*, *rteB*, *rteC*	Whittle *et al*. (2002b)
BT4001ΩQAB	*B. thetaiotaomicron* containing regulatory CTnDOT genes, *tetQ*, *rteA*, *rteB*	Whittle *et al*. (2002b)
BT4001ΩQA	*B. thetaiotaomicron* containing regulatory CTnDOT genes, *tetQ*, *rteA*	G.-R. Wang (unpublished)
BT4007ΩrecA	BT4001 with insertional disruption of *recA*	Cooper *et al*. (1997)
BT4104	BT4100 containing CTnERL	Li *et al*. (1995)
BT4007	BT4001 containing CTnDOT	Shoemaker *et al*. (1989)
BT4007ΩrteB	Chromosomal disruption of *rteB* in BT4007	Moon *et al*. (2005)
BT4001pRteC	BT4001 containing constitutively expressed RteC plasmid	Moon *et al*. (2005)
Plasmids		
pGFK154	pGERM with 1 kb gene between BT4742 and BT4743 of CTn4-bt	This study
pGRK155	pGERM with 1.1 kb gene between BT0075 and BT0076 of CTn1-bt	This study
pGFK156	pGERM with 1.1 kb gene between BT2282 and BT2283 of CTn2-bt	This study
pGFK156	pGERM with 1.1 kb gene between BT2606 and BT2607 of CTn3-bt	This study
pGFK162	pGERM with 0.6 kb gene between p5482-20 and p5482-21 of p5482	This study
pGFK166	pGERM with 0.4 kb gene of BT4770_traG_ in CTn4-bt	This study
pGFK167	pEPE with 1 kb gene between BT4742 and BT4743 of CTn4-bt	This study

### RNA isolation, microarray and RT-PCR analysis

Total RNA was extracted by using a protocol based by TRIzol® (Invitrogen). In order to check for the elimination of genomic DNA, RT-PCR analysis was performed using *Bacteroides* σ_70_ as an internal standard. RT-PCR products were visualized on 2% agarose gels. After removing genomic DNA, RNA was purified and concentrated by RNeasy column (Qiagen) (Moon *et al*., 2005). cDNA was generated from approximately 3 μg volumes of each RNA sample by using Superscript-II RT (Invitrogen). The RNA template was destroyed by incubation with 0.25 N NaOH for 30 min at 65°C and the sample was neutralized with 0.25 N HCl. The DNA was purified using a Qiaquick spin column (Qiagen). In order to perform hybridization of cDNA targets to each of the *B. thetaiotaomicron* GeneChips, the prepared cDNA was fragmented by DNase I and then biotinylated (Enzo-BioArray Terminal Labelling Kit). Biotinylation was confirmed by EMSA (Electromobility Shift Assay) using streptavidin as a secondary reagent. Hybridization was performed using Standard Affymetrix protocol at the Biotechnology centre in Washington University (St Louis, MO) (Sonnenburg *et al*., 2005). GeneChip data were analysed using DNA-Chip Analyser v1.3 (dChip; http://www.biosun1.harvard.edu/complab/dchip). GeneChips were normalized, and model-based expression was generated.

Due to the high cost and limited availability of the chips, the preliminary comparison was performed on only two GeneChip data sets to identify tentatively genes upregulated or downregulated in the experimental group (E) relative to the baseline group (B). The experimental group was cDNA from BT4007 (BT4001ΩCTnDOT) with Tc induction and the baseline group was the cDNA sample from BT4001 without Tc induction. Genes that showed more than sevenfold induction (E/B > 7) were selected for further investigation. The reason for choosing sevenfold for the cut-off was that Tc has been reported to stabilize some messages, an effect that is usually less than five- to sevenfold (Wei and Bechhofer, 2002).

Quantitative PCR was performed using an iQcycler (Bio-Rad). Expression of the *Bacteroides* σ_70_ gene was used as an internal standard and SYBR Green Supermix was used as a signal reporter. Reactions were performed in a 96-well microtitre PCR plate using the following final concentrations: 0.4 μM sense and antisense primers for each targeted gene, 3 μM MgCl_2_, 1 X iQ ™ SYBR® Green supermix (Bio-Rad) and 1 μl of cDNA. Cycling conditions were as follows: denaturation (95°C for 3 min), amplification and quantification [95°C for 30 s, optimal annealing temperature for each primer set for a target gene (°C) for 30 s, and 72°C for 30 s, with a single fluorescence measurement at both 53.7°C for 30 s and 72°C for 30 s segments] repeated 40 times. A melting curve programme (50–95°C with a heating rate of 0.1°C s^−1^ and continuous fluorescence measurement) and a cooling step to 50°C were used. Each sample was tested in triplicate. Data were analysed using the iQcycler analysis software (Bio-Rad). Relative quantification, which determines the changes in steady-state mRNA levels of a gene across multiple samples and provides a result relative to the levels of an internal control RNA, was used (Moon *et al*., 2005).

### Transfer of CTn1-bt, CTn2-bt, CTn3-bt and CTn4-bt

To determine if these putative CTns were mobilized in the presence of CTnERL, a CTn that is nearly identical to CTnDOT except for lacking the *ermF* region, we used a suicide vector to introduce *ermG* into non-coding regions of each cryptic element. A suicide vector containing *ermG* and a specific DNA fragment that targets a non-coding region of CTn4-bt were constructed and transformed into DH5αMCR. The vectors were transferred into *B. thetaiotaomicron* BT4100 by a triparental mating using HB101 (RP4) and BT4100 (Thy^–^) *B. thetaiotaomicron* strains (Li *et al*., 1995). The BT4100 donor strains contained various combinations of CTnERL or combinations of individual genes such as *tetQ*, *rteA*, *rteB* and *rteC.* The recipient was BT4001. Mating procedures have been described previously (Shoemaker and Salyers, 1987). Two *B. fragilis* strains were also used as non-isogenic recipients. *B. fragilis* 2553R and *B. fragilis* 638R were provided by Jeff Smith (East Carolina University, Greensboro, NC). The transfer efficiency was expressed as the number of transconjugants per recipient (Li *et al*., 1995). The transfer efficiency was performed three independent times and each sample was tested in triplicate. A range is given to indicate the variability in such assays, which is usually about 10-fold.
